# Hepatocellular Carcinoma Metastasis to the Tonsil: A Case Report of a Rare Entity

**DOI:** 10.7759/cureus.35943

**Published:** 2023-03-09

**Authors:** Paraskevi Karamitsou, James Philip Skliris, Spyridon Gougousis, Aikaterini Karamitsou, Alexandros Poutoglidis

**Affiliations:** 1 Department of Otorhinolaryngology - Head and Neck Surgery, 'G. Papanikolaou' General Hospital, Thessaloniki, GRC; 2 Department of Pathology, 'G. Papanikolaou’ General Hospital, Thessaloniki, GRC; 3 Department of Otorhinolaryngology - Head and Neck Surgery, 'G. Papanikolaou’ General Hospital, Thessaloniki, GRC; 4 Fourth Department of Surgery, School of Medicine, Aristotle University of Thessaloniki, ‘G. Papanikolaou’ General Hospital, Thessaloniki, GRC

**Keywords:** oropharynx, lesion, tonsil, extra-hepatic metastasis, hepatocellular carcinoma

## Abstract

Hepatocellular carcinoma (HCC) is the most dominant malignant neoplasm of the liver and constitutes the majority of all primary malignancies. Most reported cases of HCC occur in the developing world and are mainly associated with chronic hepatitis B and C viruses. Both hematogenous and lymphatic spreading is common in HCC. Patients with HCC might manifest extra-hepatic metastases and the lungs are the most common potential site of metastatic deposits. Rare sites of metastatic disease have also been described. Oropharyngeal metastases of HCC are rare and there are few reports available in the literature. We report a rare case of extra-hepatic metastasis of HCC to the right tonsil in an 84-year-old patient. The clinical appearance of metastatic oral lesions could be easily underestimated, and diagnosis of the primary tumor might delay. A biopsy of the oral lesion is important for an accurate diagnosis. Metastasis in the oral cavity and oropharynx of an HCC is usually evidence of widespread disease and predisposes to an ominous prognosis.

## Introduction

Hepatocellular carcinoma (HCC) is the most dominant malignant neoplasm of the liver and accounts for 90% of all primary malignancies. It is the fifth most common cause of cancer worldwide and the second cause of death in men following lung cancer [1]. Hepatitis B (HBV) and C (HCV) viruses, alcoholic liver disease, and non-alcoholic liver steatohepatitis/non-alcoholic fatty liver disease are the main risk factors for the development of HCC [2]. Cirrhosis caused by primary biliary cholangitis, hemochromatosis, and alpha-1 antitrypsin deficiency constitutes other less frequent risk factors. More than 70% of the cases of HCC are associated with chronic HBV and HCV [2]. Most reported cases occur in developing countries, such as Sub-Sahara Africa, South-East Asia, and China, because of the social-economical status that predisposes them to more frequent exposure to HBV and HCV. The median age of HCC diagnosis is 64 years. According to the differentiation of the tissue, the neoplasm may be well, moderately, or poorly differentiated. 30%-50% of the patients with HCC might manifest extra-hepatic metastasis [3]. Both hematogenous and lymphatic spreading is common and lungs, intra-abdominal lymph nodes, bones, and adrenal glands consist of potential sites of metastatic deposits of HCC [4]. However, the rectum, spleen, diaphragm, esophagus, pancreas, and urinary bladder have also been described as rare sites of metastatic disease. We report a rare case of extra-hepatic metastasis of HCC to the right tonsil in an 84-year-old patient.

## Case presentation

An 84-year-old, male presented to our Ear, Nose, and Throat (ENT) clinic with the chief complaint of one-month history of a lesion noticed in the apex of the right tonsil. Difficulty in swallowing with pain (dyscataposia) and loss of appetite was also reported as accompanying symptoms. The patient’s medical history included hypertension, hypothyroidism, dyslipidemia, and benign prostate hyperplasia. He was a former smoker with a referred smoking of 20 cigarettes per day for 65 years. Alcohol consumption was not mentioned.

**Figure 1:**
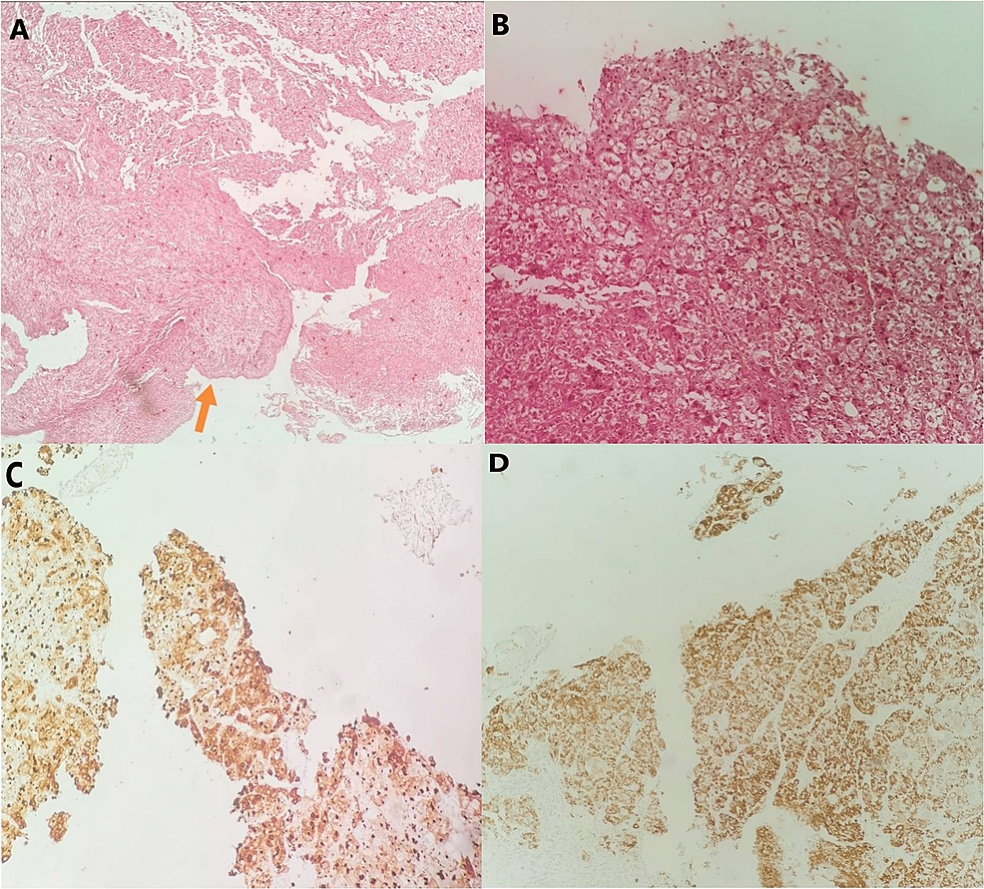
(A) Diffuse infiltration of tonsilar lamina propria from malignant cells. The overlying epithelium is visible (arrow) (hematoxylin and Eosin stain [H&E], 4x), (B) higher power illustrating the highly atypical malignant cells (H&E, 10x), (C) neoplastic cells exhibited positivity for cytokeratins (CK 8/18, 10x), (D) positivity for this marker pointed toward liver derivation (Hepatocyte Parafin 1 [HepPar1], 10x).

A thorough physical examination was performed. Clinical examination of the oropharynx revealed a soft tissue lesion in the upper lobe of the right tonsil. The lesion was friable but not tender. Neck palpation and flexible nasolaryngoscopy did not reveal any other abnormal findings. Laboratory examinations demonstrated normal values. A neck magnetic resonance imaging (MRI) was performed showing a mass in the right tonsil, measuring 13x16x22mm, with enhancement after intravenous paramagnetic contrast agent administration. Invasion of the surrounding structures or locoregional lymph node involvement was not noticed. Subsequently, a biopsy under local anesthesia was performed, concluding in the diagnosis of a poorly differentiated carcinoma, probably a metastasis from another primary tumor (Figures 1A-1D).

**Figure 2:**
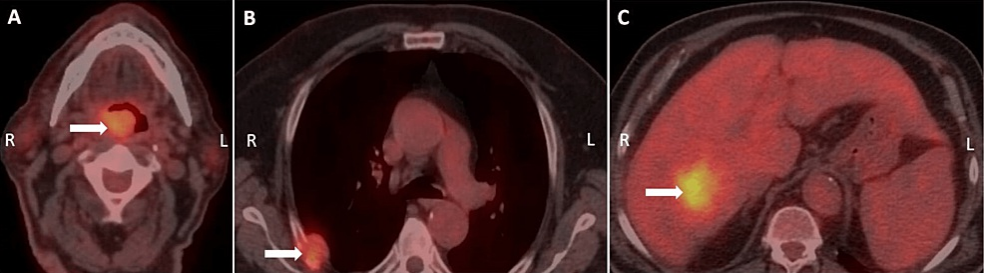
18F-FDG PET/CT. (A) A lesion in the right tonsil (arrow) with intense hypermetabolic activity, (B) a lytic lesion on the right fifth rib (arrow) with an accompanying intensely hypermetabolic soft tissue mass, and (C) a mass in segment VII of the liver (arrow) with an intense hypermetabolic activity. Higher SUVmax was recorded for the liver mass.

18F-fluorodeoxyglucose (18F-FDG) positron emission tomography/computed tomography (PET/CT) was requested to identify the primary tumor. A lesion in the middle lobe of the right lung with a maximum Standard Uptake Value (SUVmax) of 4.3, a mass in segment VII of the liver with an intense hypermetabolic activity with SUVmax of 8.0, a focal lesion to the rectosigmoid wall with SUVmax of 6.1, and a lytic lesion on the right fifth rib with an accompanying intensely hypermetabolic soft tissue mass with SUVmax of 5.0 were found (Figures 2A-2C). The liver was considered the most possible primary site because of its higher SUVmax.

The multidisciplinary team (MDT) of our hospital recommended biopsies from the abnormal lesions described in the 18F-FDG PET/CT for identification of the primary tumor. Thus, a biopsy under local anesthesia was conducted from the soft tissue mass of the chest. The histology report indicated a poorly differentiated metastatic HCC. Immunochemistry showed pan cytokeratin (AE1/AE3), cytokeratins 8 and 18 (CK 8/18), and hepatocyte-specific antigen-antibody positivity, while CD 10 expression was rarely positive. Comparative examination between this specimen and the one taken from the right tonsil demonstrated similar morphological features and immunochemistry markers, revealing that both sites are metastases of HCC. Additionally, a colonoscopy was scheduled and a polyp (diameter = 5mm) was removed. The histology report indicated a tubular adenoma in the colon.

A gastroenterological examination was performed. A computed tomography (CT) of the abdomen demonstrated liver cirrhosis, a 40x30x20mm mass in segment VII of the liver, and portal vein thrombosis. Additional laboratory examinations were requested; the patient was detected to be positive for HBV surface antigen, the level of HBV-DNA was <10IU/mL, and the a-fetoprotein (AFP) level was 43ng/mL (normal range 0.89-8.78ng/mL), while the liver function tests were within normal range.

The patient received three cycles of chemotherapy with paclitaxel and carboplatin, followed by immunotherapy with atezolizumab and bevacizumab. The duration of the treatment was fifteen weeks. He died six months after the end of the medical treatment.

## Discussion

Metastatic carcinoma of the oropharynx is rare, and it accounts for approximately 1% of all malignant tumors [5]. The most common sources of metastatic lesions to the oral mucosa are breast cancer for women and lung cancer for men [6]. Oral cavity and oropharyngeal metastases of HCC are uncommon and only a few cases have been reported in the literature [7]. Metastasis in the oral cavity is usually evidence of widespread disease [6]. In such cases, the prognosis after the assessment of the lesion in the oral cavity is poor, due to the high progression of the disease. Additionally, oral and oropharyngeal metastases are rarely the first manifestation of the HCC [6, 8], while in a significant proportion of patients (20.4%) oral mucosa metastases were manifested before the primary tumor in the lung, kidney, breast, skin, bone, or stomach was diagnosed [6].

Pain, difficulty in swallowing, dyspnea, bleeding, and loss of appetite are some of the reported symptoms in patients with oral or oropharyngeal metastases of HCC [6,9]. Clinical examination of the oral cavity and oropharynx might reveal an easily bleeding, ulcerated, or rapidly growing lesion [10].

Metastases in the oral cavity and oropharynx are thought to be associated with spreading either hematogenous or lymphatic [6]. Batson et al. [11] proposed the valve-less vertebral venous plexus as a mechanism for bypassing filtration through the lungs. The tumor cells through blood circulation reach the oral cavity and oropharynx and they might be entrapped in the rich capillary network of the chronically inflamed oral mucosa.

According to the literature, the five-year overall survival for HCC is 18% [12]. Tumor size, tumor differentiation, liver function, presence of metastases, tumor extension, and high levels of AFP are some factors that predispose to an ominous prognosis [13]. There is no clear evidence for survival in patients with oral metastases by HCC, in the few reported cases in the literature [7]. According to Kanazawa et al. [14], the mean overall survival rate after oral metastases of HCC is 21 weeks.

## Conclusions

The oropharynx is not a common site of metastasis of HCC. The clinical appearance of a metastatic oral lesion might even resemble a benign one. Thus, the clinical diagnosis could be misleading and might cause a delay in the diagnosis of a primary tumor. A timely diagnosis requires high clinical suspicion. The role of biopsy is indisputable in primary tumor identification. Oral and oropharyngeal metastases usually occur in advanced stages, and they are evidence of widespread disease. As a result, patients presenting with oral or oropharyngeal metastases usually have a poor prognosis.

## References

[1] Ferlay J, Soerjomataram I, Dikshit R (2015). Cancer incidence and mortality worldwide: sources, methods and major patterns in GLOBOCAN 2012. Int J Cancer.

[2] Ioannou GN, Splan MF, Weiss NS, McDonald GB, Beretta L, Lee SP (2007). Incidence and predictors of hepatocellular carcinoma in patients with cirrhosis. Clin Gastroenterol Hepatol.

[3] Natsuizaka M, Omura T, Akaike T (2005). Clinical features of hepatocellular carcinoma with extrahepatic metastases. J Gastroenterol Hepatol.

[4] Harding JJ, Abu-Zeinah G, Chou JF (2018). Frequency, morbidity, and mortality of bone metastases in advanced hepatocellular carcinoma. J Natl Compr Canc Netw.

[5] Meyer I, Shklar G (1965). Malignant tumors metastatic to mouth and jaws. Oral Surg Oral Med Oral Pathol.

[6] Hirshberg A, Leibovich P, Buchner A (1993). Metastases to the oral mucosa: analysis of 157 cases. J Oral Pathol Med.

[7] Pires FR, Sagarra R, Corrêa ME, Pereira CM, Vargas PA, Lopes MA (2004). Oral metastasis of a hepatocellular carcinoma. Oral Surg Oral Med Oral Pathol Oral Radiol Endod.

[8] Llanes F, Sanz-Ortega J, Suarez B, Sanz-Esponera J (1996). Hepatocellular carcinomas diagnosed following metastasis to the oral cavity. Report of 2 cases. J Periodontol.

[9] Nadkarni S, Patkar S, Acharya R, Shah A, Patel S, Parray A, Goel M (2020). Hepatocellular carcinoma metastasis to the buccal mucosa masquerading as oral cavity malignancy: Case report of a rare entity. Ann Hepatobiliary Pancreat Surg.

[10] Carroll O, Krolls SO, Mosca NG (1993). Metastatic carcinoma to the mandible: report of two cases. Oral Surg Oral Med Oral Pathol.

[11] Batson OV (1940). The function of the vertebral veins and their role in the spread of metastases. Ann Surg.

[12] Jemal A, Ward EM, Johnson CJ (2017). Annual report to the nation on the status of cancer, 1975-2014, featuring survival. J Natl Cancer Inst.

[13] Matsumoto Y, Suzuki T, Asada I, Ozawa K, Tobe T, Honjo I (1982). Clinical classification of hepatoma in Japan according to serial changes in serum alpha-fetoprotein levels. Cancer.

[14] Kanazawa H, Sato K (1989). Gingival metastases from primary hepatocellular carcinoma: report of a case and review of literature. J Oral Maxillofac Surg.

